# Cerebral Metabolic Network in Patients With Anti-*N*-Methyl-D-Aspartate Receptor Encephalitis on 18F-FDG PET Imaging

**DOI:** 10.3389/fnins.2022.885425

**Published:** 2022-04-29

**Authors:** Gan Huang, Mei Xin, Yong Hao, Shuwei Bai, Jianjun Liu, Chenpeng Zhang

**Affiliations:** ^1^Department of Nuclear Medicine, Ren Ji Hospital, Shanghai Jiao Tong University School of Medicine, Shanghai, China; ^2^Department of Neurology, Ren Ji Hospital, Shanghai Jiao Tong University School of Medicine, Shanghai, China

**Keywords:** Autoimmune disease, Anti-*N*-methyl-D-aspartate receptor encephalitis, ^18^F fluorodeoxyglucose, positron emission tomography, brain connectivity, graph theory

## Abstract

**Background:**

Anti-*N*-methyl-D-aspartate receptor (NMDAR) encephalitis is the most common autoimmune encephalitis (AE), and the prognosis may significantly be improved if identified earlier and immune-related treated more effectively. This study evaluated the brain metabolic network using fluorodeoxyglucose positron emission tomography (FDG PET).

**Material and methods:**

FDG PET imaging of patients with NMDAR encephalitis was used to investigate the metabolic connectivity network, which was analyzed using the graph theory. The results in patients were compared to those in age- and sex-matched healthy controls.

**Results:**

The hub nodes were mainly in the right frontal lobe in patients with NMDAR encephalitis. The global and local efficiencies in most brain regions were significantly reduced, and the shortest characteristic path length was significantly longer, especially in the temporal and occipital lobes. Significant network functions of topology properties were enhanced in the right frontal, caudate nucleus, and cingulate gyrus. In addition, the internal connection integration in the left cerebral hemisphere was poor, and the transmission efficiency of Internet information was low.

**Conclusion:**

The present findings indicate that those characteristic and connections of metabolic network were changed in the brain by graph theory analysis quantitatively, which is helpful to better understand neuropathological and physiological mechanisms in patients with anti-NMDAR encephalitis.

## Introduction

Anti-*N*-methyl-D-aspartate receptor (NMDAR) encephalitis is a common autoimmune encephalitis (AE) that frequently presents with various complex psychotic symptoms, amnesia, seizures, and movement disorders (Abboud et al., 2021). The pathological changes might involve NMDAR-Ab, which changes intrinsic cortical connections and neuronal population dynamics (Rosch et al., 2018; Dalmau et al., 2019). To the best of our knowledge, most studies in the literature have focused on changes in brain networks using magnetic resonance imaging (MRI) and electroencephalography (EEG) in patients with encephalitis (Peer et al., 2017; Xu et al., 2017; Heine et al., 2018; Symmonds et al., 2018; Bauer et al., 2020; Li et al., 2021; Wang et al., 2021), while the use of ^18^F-fluorodeoxyglucose (FDG) positron emission tomography (PET)/computed tomography (CT) has not been reported before, which is earlier for recognition and more sensitive than MRI (Novy et al., 2016; Yuan et al., 2016; Wei et al., 2020; Seniaray et al., 2021). Metabolic brain network analysis based on graph theory has proved useful in analyzing brain activity changes and is widely used in neuropsychological imaging (Huang et al., 2020; Rajagopalan and Pioro, 2020). In particular, metabolic network analysis using FDG PET provides functional interregional connectivity information and has been reported to provide differential diagnostic capabilities for mental diseases (Huang et al., 2018). To the best of our knowledge, our study is the first to focus on changes in the brain metabolic network in patients with anti-NMDAR encephalitis; we quantified the internal working mechanisms of complex brain networks, explored the network topology attributes, reproduced the information transmission mode between different brain regions, and detected specific changes in functional or structural connectivity in the brain of patients with anti-NMDAR encephalitis.

## Materials and Methods

### Data Acquisition

A retrospective analysis was performed on patients with anti-NMDAR encephalitis who underwent PET/CT examination in our center from August 2012 to September 2020. The modified Rankin Scale (mRS) scores of these patients were assessed at the time of conducting the PET/CT examination. Seventeen age- and sex-matched healthy subjects with normal physical examination and without a history of neurological or psychological disease were selected as the control group. All patients were signed informed consent before PET/CT examination.

### Imaging Protocol

Before the examination, all patients fasted for more than 6 h, and their blood glucose concentration was <7.8 mmol/L before injection of ^18^F-FDG.

PET acquisition started approximately 45 ± 10 min after intravenous administration of approximately 3.7 MBq/kg. Patients waited and scanned in a relatively quiet and bright environment without an eye mask while undergoing PET-CT.

PET studies were performed in 15 patients using a Siemens mCT scanner (Siemens Healthcare, Knoxville, Tennessee, USA) and two patients using a uMI®780 scanner (United Imaging, Shanghai, China). These PET images were reconstructed using an ordered subset expectation maximization (OSEM) iterative reconstruction method.

To perform consistent and effective analyses, we eliminated morphological differences between individual brains. First, the Statistical Parametric Mapping (SPM12) software package in MATLAB (Mathworks Inc., Natick, MA, USA) was applied, which linearly registered the images into Montreal Neurological Institute (MNI) space using the MNI's PET.NII template. The brain mask in SPM was then used to remove the skull and scalp, and the cerebral cortex was segmented. Finally, an 8-mm full-width at half-maximum isotropic Gaussian kernel was used for spatial smoothing to improve the signal-to-noise ratio. An automated anatomical labeling 90 atlas (AAL90) template image verified by Tzourio-Mazoyer et al. (Tzourio-Mazoyer et al., 2002) was used to complete region segmentation. The brain was divided into 90 anatomical regions of interest (45 brain regions per hemisphere), and the average intensity value of each region was calculated to represent the regional brain metabolic rate of glucose.

### Network Construction

The construction of a metabolic network is mainly used to define network nodes and connection edges between nodes. In this study, the brain was defined as having 90 nodes using the AAL90 template provided by the MNI. For the definition of edge, the Pearson correlation coefficient (*r*) between the metabolic intensity values of brain regions was calculated as the connection of the metabolic network, and the concept of such connection was introduced in Lerch et al.'s study (Lerch et al., 2006). The formula for the degree of correlation between any two network nodes is given as follows:


(1) $$\begin{array}{l} r=\frac{\overset{n}{\underset{i=1}{\sum }}\left(x_{i}-\bar{x}\right)\left(y_{i}-\bar{y}\right)}{\sqrt{\overset{n}{\underset{i=1}{\sum }}{\left(x_{i}-\bar{x}\right)}^{2}}\sqrt{\overset{n}{\underset{i=1}{\sum }}{\left(y_{i}-\bar{y}\right)}^{2}}} \end{array}$$

where *x*_*i*_ represents the metabolic intensity value of the i^th^ sample of a certain brain region, and $\bar{x}$ represents the mean metabolic intensity of this brain region; *y*_*i*_ represents the metabolic intensity value of the i^th^ sample of another brain region, and $\bar{y}$ represents the mean metabolic intensity of this brain region; and n is the number of samples in each group.

### Brain Network Analysis

After the network was constructed, the functional brain network of the healthy control group and the encephalitis patient group was calculated using graph theory. We calculated the following global parameters: (1) average characteristic path length (L), which is the average of the shortest path length between one node and all nodes; (2) mean global efficiency (GlobE), which is the inverse shortest path length; (3) average local efficiency (LocE), which is the average LocE of all nodes; and (4) average clustering coefficient (C), which is the average node fraction of the degree of possible connection number in its neighborhood.

To evaluate the area network, we calculated the following node parameters for each node: (1) node path length, which is the shortest path length between the node and all other nodes; (2) clustering coefficient of nodes, which is the ratio of clustering degree in its neighborhood to the number of possible connections between nodes; (3) the GlobE of nodes, which is the average inverse shortest path length from one node to all other nodes; (4) the LocE of nodes, which is the average inverse shortest path length from a node to its neighborhood; and (5) node centrality, which is a statistical index used to describe the role and status of network nodes and is usually used to determine the candidate hub nodes in the network. This study used the open-source graph analysis software Braph (http://braph.org/) (Mijalkov et al., 2017) and GRETNA (https://www.nitrc.org/projects/gretna/) (Wang et al., 2015) network characteristic parameters. To determine the statistical significance of the difference in network parameters between the healthy control group and the encephalitis patient group, we used 1,000 repeated nonparametric permutation tests and corrected for multiple comparisons using the null hypothesis false discovery rate (FDR) test; *P* < 0.05 was considered statistically significant. BrainNet Viewer (https://www.nitrc.org/projects/bnv/) was used to visualize the display toolkit.

Node centrality is a very important measurement index based on graph theory that reflects the relative importance of nodes in the network and helps identify the hub nodes in a network. In this study, node centrality BCi> (mean + SD) was defined as the hub node, where MEAN was the mean of the medial centrality of all nodes in the group, and SD was its standard deviation (Gong et al., 2009).

## Results

### Parametric Analysis of Whole Brain Network

Seventeen patients were included in the analysis, and their characteristics, such as median age (35 years; range, 14–67 years; 10 men) and other clinical information, are summarized in Table 1. There were 14 (82.35%) patients with good status (mRS scores of 0–2) and 3 (17.65%) patients with relatively poor status (mRS score of 3), respectively. No one got a score higher than 3. A total of 1/7 women had ovarian teratoma when screened using PET/CT in 21 days, who had mental status changes and seizures for half a month, accompanied by cognitive impairment and speech disorders. In addition, 4/17 (23.53%) patients had viral encephalitis before being diagnosed. We divided patients into three groups for subgroup based on the interval time of examination defined as the onset of the symptoms to the PET examination (≤1 month, 2–3 months, and >3 months). The number of patients was 5/17 (29.41%), 7/17 (41.18%), and 5/17 (29.41%), while the median age was 24, 39, and 35 years, and the number of male patients was 2/5 (40%), 6/7 (85.71%), and 2/5 (40%) for each stage.

**Table 1:** Summary of clinical data of all patients.

**Characteristics**	**Patients**
Gender (M/F)	10/7
Age (years): median ± standard deviation	35 ± 16.68
Mean duration from manifestation until PET (months)	1.73 ± 1.51
Tumor	Ovarian teratoma:
	*n =* 1;
Typical symptoms of AE (patient)	
Abnormal behaviors or cognitive dysfunction	13
Speech dysfunction	9
Seizure	11
Movement disorder, dyskinesias, or rigidity/abnormal postures	13
consciousness impairment	7
Autonomic dysfunction or central hypoventilation	9
Modified Rankin Scale scores	
1	3
2	11
3	3
Based on the time from symptom onset to the examination	
≤ 1 month	5
2–3 months	7
>3 months	5

**Figure 1:**
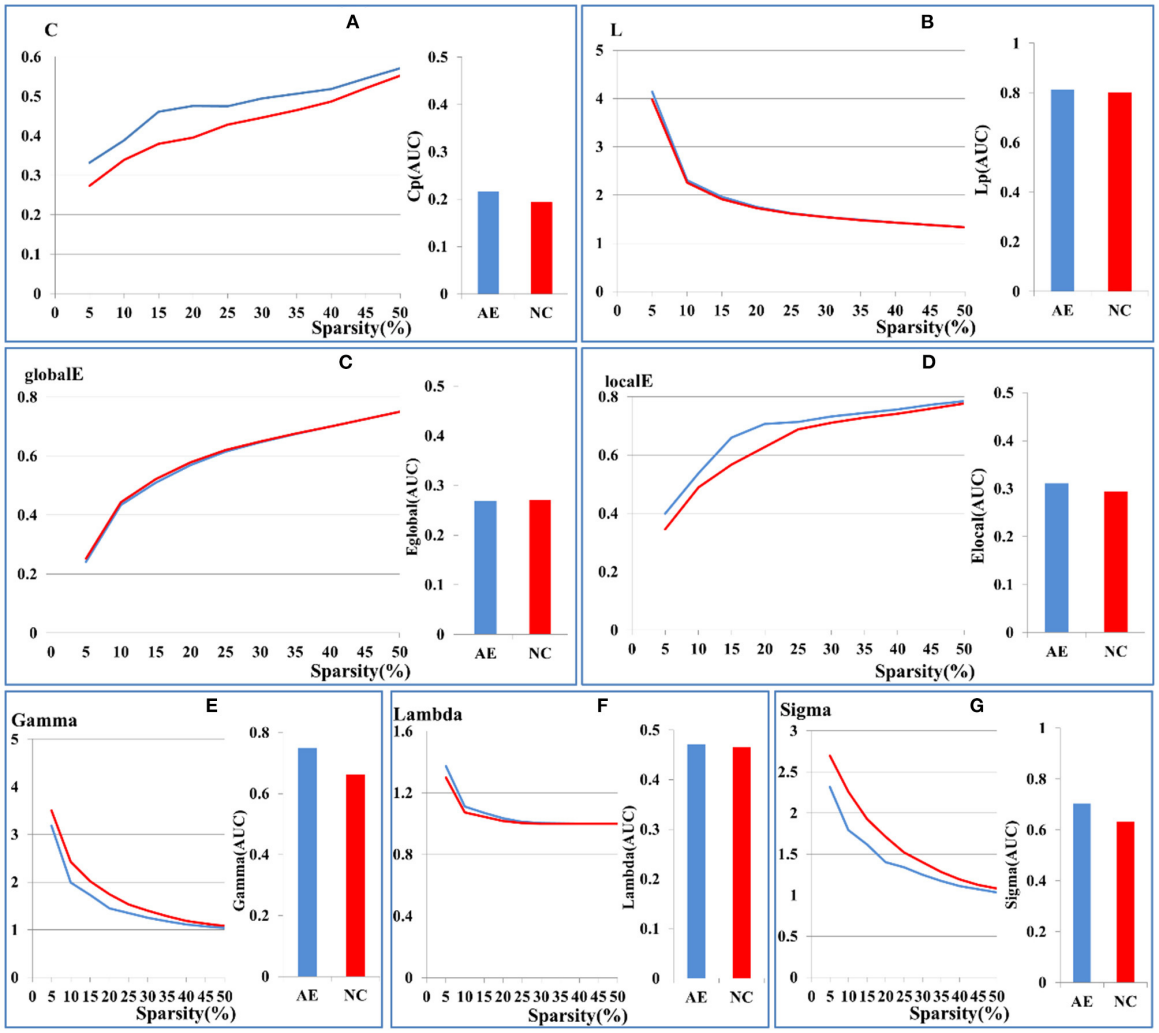
Comparison of global brain network parameters between patients with anti-NMDAR encephalitis and health controls. **(A)** clustering coefficient, **(B)** shortest characteristic path length, **(C)** global network efficiency, **(D)** local network efficiency. Sigma values of the two groups in the range of 5–50% sparsity was calculated by Gamma **(E)**, Lambda **(F)**, Sigma **(G)**. The X-axis coordinates represent sparsity thresholds ranging from 5% to 50%. Results from patients with anti-NMDAR encephalitis are in blue, and those from healthy controls are in red. The bar chart shows the AUC values of the two groups.

There were no significant differences in the small-world network attributes for the whole brain network parameters between AE and the control groups (*p* > 0.05), which included the clustering coefficient, shortest characteristic path length, or global and local network efficiency, as shown in Figures 1A–D. Both groups had small-world network attributes, in which gamma >> 1, lambda ~ 1, and sigma > 1, as shown in Figures 1E–G. The results of our study indicate that the analysis of functional connectivity was feasible for the brain metabolic network of patients with anti-NMDAR encephalitis (Schmid et al., 2020).

### Hub Nodes

**Figure 2:**
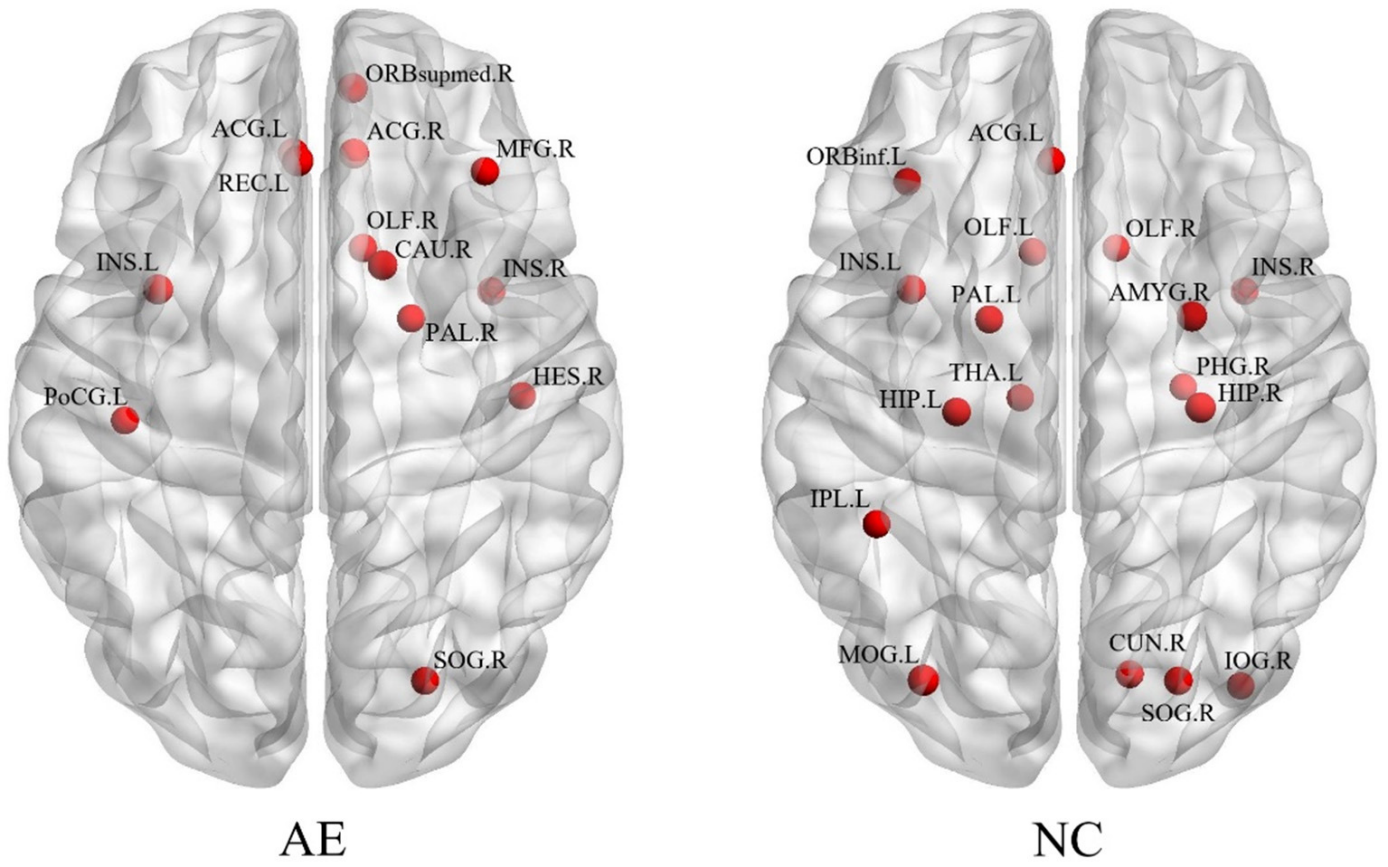
The brain regions with different network parameters in the encephalitis patient group and healthy control group were different.

Figure 2 shows the distribution of brain hub nodes in patients with anti-NMDAR encephalitis and healthy controls (13 vs. 17 brain hub nodes). The bilateral insula (ins. L, ins. R), left anterior cingulate and paracingulate gyrus, right occipital gyrus, and right olfactory cortex were common core nodes in patients with encephalitis and healthy controls. In addition, the core nodes of the left hemisphere in patients were significantly reduced compared to the healthy controls. Moreover, most of the hub nodes of the patients' group were concentrated in the right frontal lobe, while there were scattered in the whole brain for the healthy controls.

### Network Analysis of Local Brain Regions

**Figure 3:**
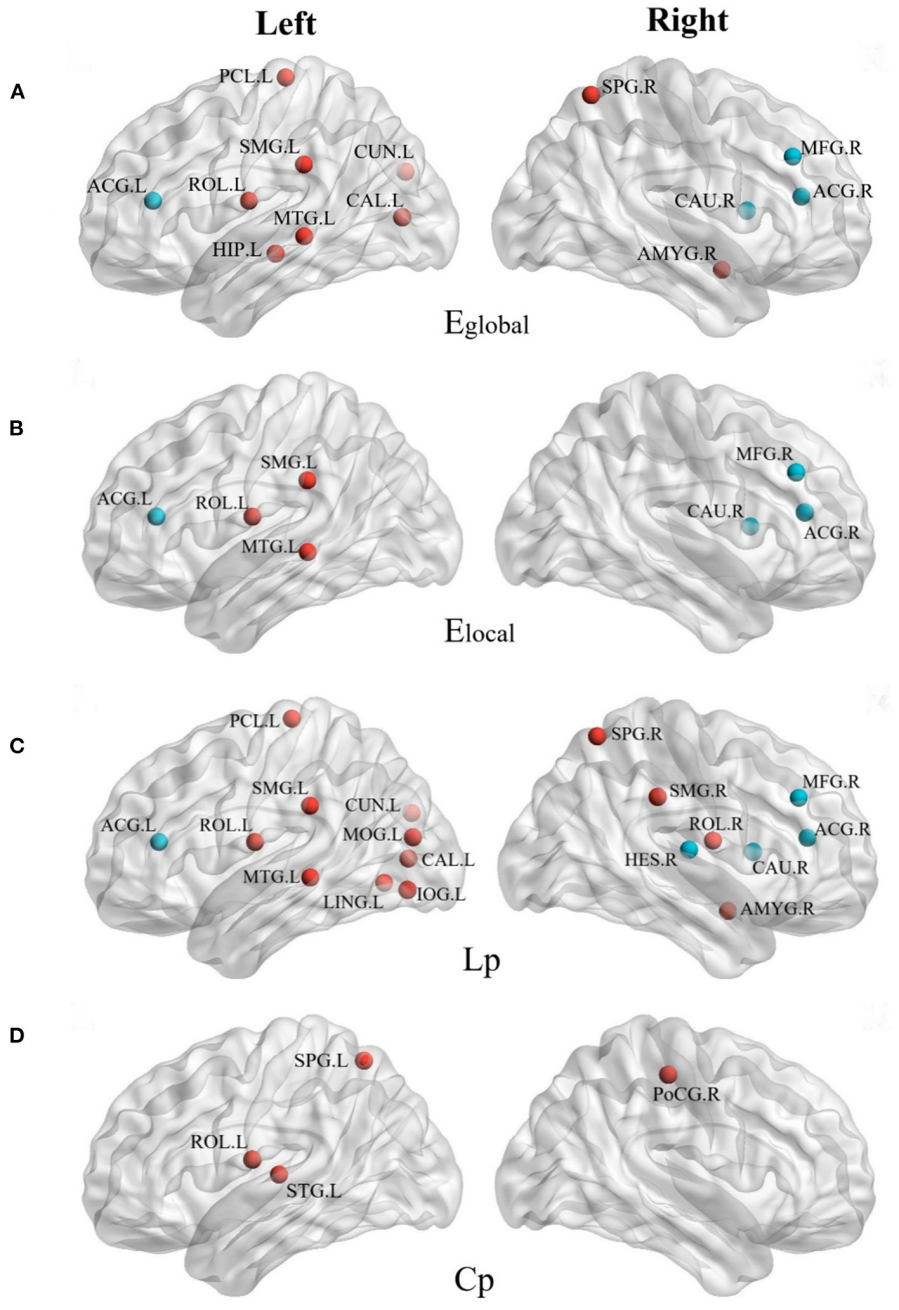
The brain regions with different network parameters in the encephalitis patient group and the normal control group were different (*P* < 0.05, red indicates that the network topology attribute was superior in NC, and blue indicates that the network topology attribute was superior in AE). **(A)** Brain regions with significant differences in global network efficiency (Eglobal) between NC group and AE group; **(B)** Brain regions with significant differences in local network efficiency (Elocal) between NC group and AE group; **(C)** The brain regions with shortest characteristic path length (Lp) significantly different between NC group and AE group; **(D)** Brain regions with significant clustering coefficient of path nodes (Cp) differences between NC group and AE group.

Figure 3 shows the distribution of brain regions with differences in network parameters between the anti-NMDAR encephalitis group and the healthy control group, and the temporal and occipital lobes were the majority regions of significant differences with network characteristic parameters.

Compared with the right cerebral hemisphere, the regions of the left cerebral hemisphere were significantly reduced in the global and local efficiencies and the shortest characteristic path length was significantly longer, especially in the Left Supramarginal gyrus (SMG.L), left middle temporal gyrus (MTG.L), and Left Rolandic operculum (ROL.L). The global and LocE and characteristic path length of the bilateral middle frontal gyrus (i.e., MFG.L and MFG. R), right caudate nucleus (CAU.R), and bilateral anterior cingulate/paracingulate gyrus (i.e., ACG.L and ACG.R) of the anti-NMDAR encephalitis group were better than those of the healthy control group, which were enhanced in the network.

### Brain Laterality Analysis

**Figure 4:**
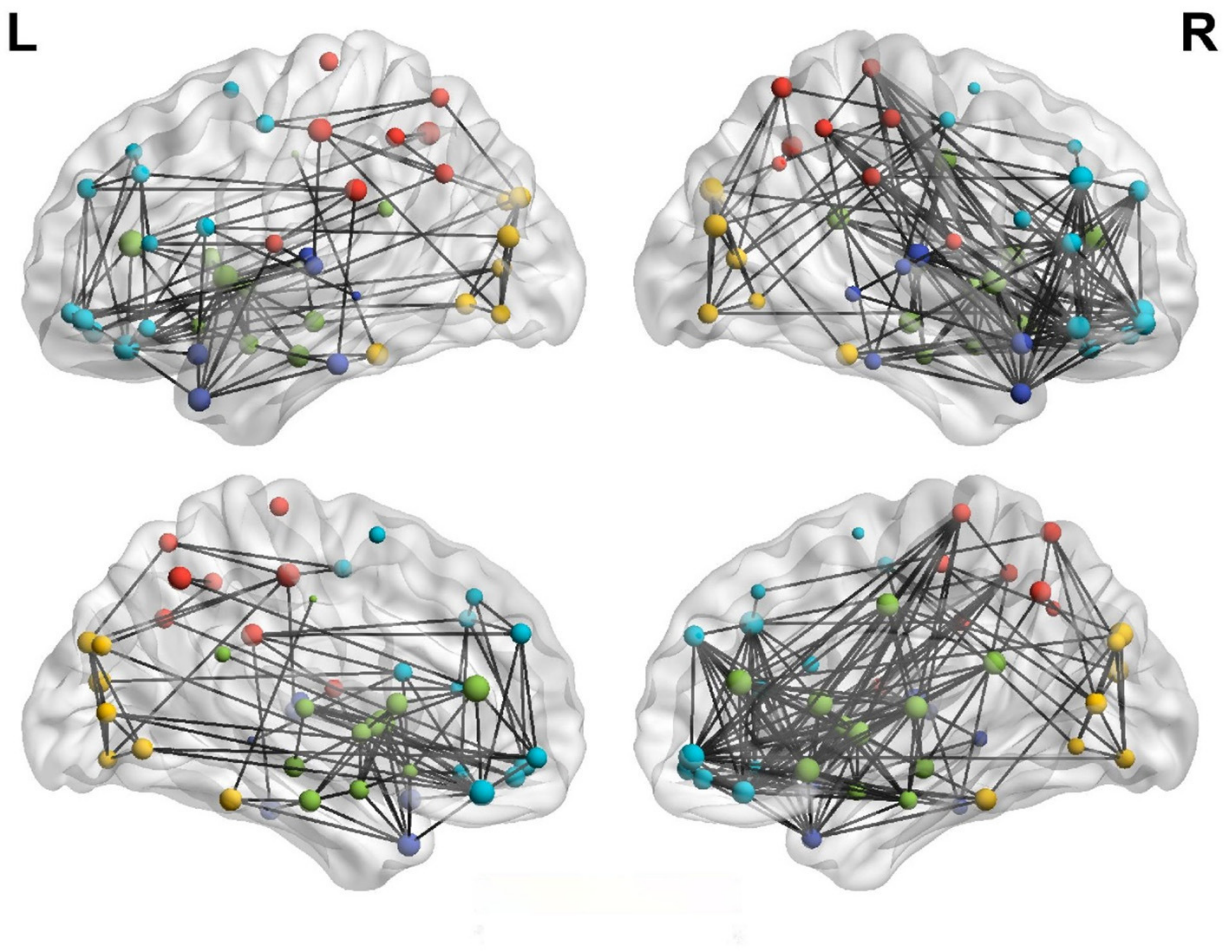
Lateral hemispheric connectivity in patients with anti-NMDAR encephalitis. Pearson correlation coefficient *R*>0.5 was the effective connection threshold, and the connection map of cerebral hemispheres was visualized in 3D view. (Dark blue is in the temporal lobe, light blue is the frontal lobe, red is the parietal lobe, yellow is the occipital lobe, and green is the limbic system).

Figure 4 shows the left and right hemispheric connectivity of patients with anti-NMDAR encephalitis using a binary matrix of predetermined threshold values. To reduce the display complexity of the networks in the left and right hemispheres, a connection correlation coefficient of *R* > 0.5 was selected to identify effective connections. The left cerebral hemisphere in patients with anti-NMDAR encephalitis showed significant disconnect compared to the right cerebral hemisphere, especially in the parietal lobe (PCL.L), frontal lobe (SFGmed.R and MFG.R), temporal lobe (TPOmid.R), and limbic brain regions. The left hemisphere has fewer direct connections between the parietal lobe and frontal and limbic regions, in contrast to the right hemisphere.

**Figure 5:**
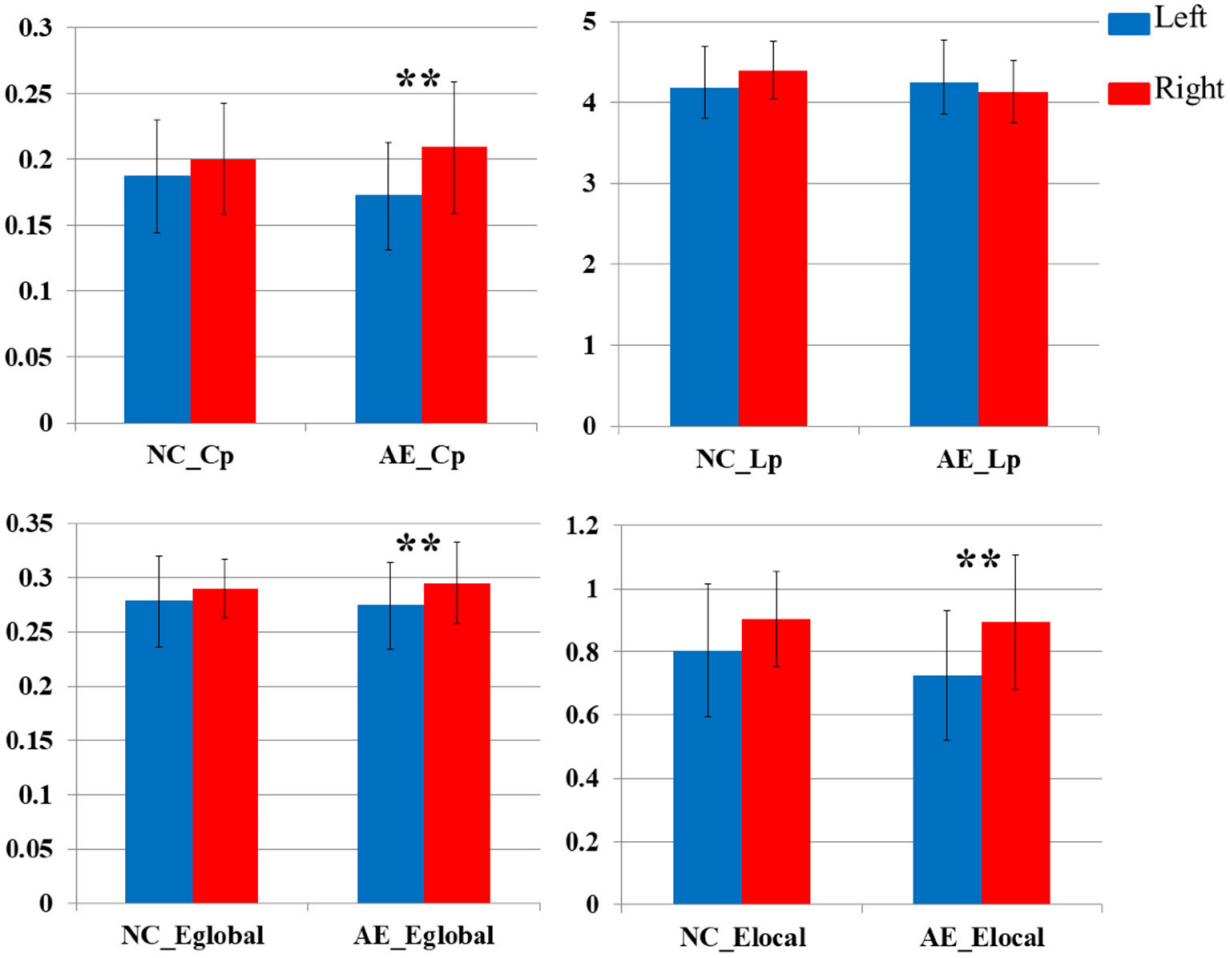
Comparative analysis of network parameters between left and right hemispheres in patients with anti-NMDAR encephalitis and healthy controls (**P < 0.001).

Figure 5 illustrates the asymmetry of the left and right hemispheres in patients with anti-NMDAR encephalitis. There were significant differences in the clustering coefficient and global and LocE between the left and right cerebral hemispheres in patients with anti-NMDAR encephalitis (*p* < 0.001), and the network information transmission efficiency of the left cerebral hemisphere was significantly lower than that of the right cerebral hemisphere. There were no significant differences in the healthy control group.

## Discussion

Previous studies have showed that the changes in FDG PET appeared earlier and had a higher rate of identifying abnormalities in patients with NMDAR encephalitis than that of MRI (Novy et al., 2016; Wei et al., 2020; Seniaray et al., 2021). Structural MRI has been shown to reveal connectivity alterations that correlate with clinical features (Peer et al., 2017); however, the pattern of FDG PET connectivity abnormalities has not been reported, as far as we know. We report the alterations in functional connectivity on FDG PET in patients with anti-NMDAR encephalitis using the graph theory analysis method.

For the first time as far as we know, we found that the right frontal lobe was mainly concentrated areas of hub nodes for patients with anti-NMDAR encephalitis, besides the insula was a hub node and is consistent with other studies (Li et al., 2021). Given that the left hemisphere is the dominant functional area in health control, we proposed that phenomenon might be a compensatory change for the impaired left hemisphere in patients and needs to be proved by large-scale data in the future. Second, we found alterations in functional connectivity and network characteristic for regional network organization, especially in the occipital and temporal lobes which were the majority regions with significant reduction and weakening. The results were similar to other studies on metabolic patterns and MRI (Peer et al., 2017; Probasco et al., 2018; Miao et al., 2020; Li et al., 2021) and suggested similarities in the pathological process leading to abnormal functional and structural changes. The disruption of the network related to clinical symptoms can improve the understanding of the neuropathological mechanisms, e.g., the disconnections between the network and the cognitive impairment (Lin et al., 2019). Third, we identified the caudate nucleus and cingulate gyrus in patients were enhanced in the network function. This is consistent with other studies by metabolic analysis (Novy et al., 2016; Yuan et al., 2016), who proposed this difference may be due to the regional glucose utilization and increased metabolism of the ketamine button. Fourth, we found a significant left–right asymmetry in network efficiency, especially for the poorer connection integration and transmission efficiency in the left hemisphere. This was potentially consistent with neuropathological findings that the asymmetry of NMDA receptors is regulated and lateralized by the unilateral distribution of NR1 and NR2B subunits (Peyvandi Karizbodagh et al., 2021). Fifth, others had found that functional connectivity within the hippocampal was significantly reduced on MRI (Finke et al., 2013; Peer et al., 2017); however, our results showed that the bilateral hippocampus was no longer as hub nodes and only the connection of left hippocampal was significantly reduced in our FDG PET study. The results need to be validated by the application of PET/MR simultaneously in the future.

In sum, we speculated that the possible mechanisms of changes in the metabolic network for anti-NMDAR encephalitis might be as follows: the dominant functional and network efficiency of the left hemisphere is impaired, especially in the left frontal lobe which carried out higher mental processes, such as speaking meaningfully and movement fluency. The failure of the connectivity and characteristic for network organization between occipital and temporal lobes resulted in a clinical symptom, such as memory loss and deliration, and affected hippocampal latterly. Meanwhile, the right frontal lobe, cingulate gyrus, and caudate nucleus might be activated as compensatory, especially for the connected short-term memory tasks, planning, and attention. These findings may be useful in understanding the pathophysiological basis of clinical processes and FDG PET manifestations. In addition, it may be useful in assisting quickly clinical diagnosis which provides immune-related therapies earlier and significantly improves prognosis. The results need to be validated by the application in the future.

The limitations of this study are as follows. First, the sample size of this study (17 cases) was limited. The results need to be validated by studies with a larger sample size. Second, it was a cross-sectional retrospectively study. The patients were at different stages of disease and their brain metabolic network was changed as the disease progresses, e.g., the cognitive functions showed differences to some extent (Mckeon et al., 2018). More longitudinal studies involving different stages are required. Third, there are some disputes among the results of the single-modal image of graph theory. We hope that it will be improved by the widespread application of PET/MR further, and multimodal image data can be used to reveal abnormalities in brain structure and function simultaneously.

## Conclusion

This study conducted a multiparameter quantitative evaluation of network changes in the brain regions of patients with anti-NMDAR encephalitis using a graph theory analysis method. The detected metabolic brain network abnormalities help improve the understanding of the neuropathological and physiological mechanisms of patients with anti-NMDAR encephalitis.

## Data Availability Statement

The raw data supporting the conclusions of this article will be made available by the authors, without undue reservation.

## Ethics Statement

The studies involving human participants were reviewed and approved by Shanghai Jiao Tong University School of Medicine, Renji Hospital Ethics Committee. Written informed consent to participate in this study was provided by the participants' legal guardian/next of kin.

## Author Contributions

Material preparation and data collection were performed and the first draft of the manuscript was written by CZ and GH. Analysis was performed by CZ, GH, YH, and SB. All authors read and approved the final manuscript, contributed to the study conception and design, and commented on previous versions of the manuscript.

## Funding

This work was supported by the Shanghai Aging, Women and Children's Health Research Program (2020YJZX0107) and the Clinical Research Innovation Cultivation Fund of Renji Hospital (PYIII20-04).

## Conflict of Interest

The authors declare that the research was conducted in the absence of any commercial or financial relationships that could be construed as a potential conflict of interest.

## Publisher's Note

All claims expressed in this article are solely those of the authors and do not necessarily represent those of their affiliated organizations, or those of the publisher, the editors and the reviewers. Any product that may be evaluated in this article, or claim that may be made by its manufacturer, is not guaranteed or endorsed by the publisher.

## Abbreviations

NMDAR: anti-*N*-methyl-D-aspartate receptor
FDG: 18F-fluorodeoxyglucose
AE: autoimmune encephalitis.
